# Temperature and salinity vertical profiles (CTD) data in the Ría de Vigo and adjacent shelf collected in the Radiales program, 1987–2020

**DOI:** 10.1016/j.dib.2023.109626

**Published:** 2023-10-04

**Authors:** Pablo Otero, Águeda Cabrero, Fernando Alonso-Pérez, Gerardo Casas, Jesús Gago, Enrique Nogueira

**Affiliations:** Centro Oceanográfico de Vigo, Centro Nacional Instituto Español de Oceanografía (IEO, CSIC), Subida a Radio Faro 50, Vigo 36003, Spain

**Keywords:** Seawater, Potential density, Oceanography, Northwest Iberia, Spain

## Abstract

The Galician rías and their adjacent continental shelf form part of the northern boundary of the Canary Current upwelling system (CanCUS), one of the world's major eastern boundary upwelling ecosystems (EBUEs). During summer, prevailing northerly winds export surface water offshore, allowing deeper, cooler, nutrient-rich water to rise, creating a fertilizing effect on coastal waters that support valuable fisheries and aquaculture economy. This data article describes a time series of hydrographic data collected on a biweekly to monthly frequency from August 1987 to September 2020 in the interior of the Ría de Vigo (one of the aforementioned Galician rías) and its adjacent shelf. This monitoring effort results in the longest sampling series in the area up to 2020, providing high value for understanding the connectivity processes between the coastal embayment and the adjacent shelf, changes in ocean climate, as well as ecosystem structure and functioning. Data were collected with vertical pressure, temperature and conductivity profilers, varying the profiler instrument over time (MARK III, SBE 9 Plus, SBE 19, SBE 25). Data were collected at four stations with depths ranging from 29 m to 148 m, although only two of these stations cover the full temporal range of the monitoring program. Due to the temporal extent of the sampling, the data have been processed with different techniques and by different technicians throughout the duration of the monitoring program. To ensure data consistency and increase data reusability, all data have been now reprocessed under the same criteria, quality-controlled, and unified in this dataset. The dataset in both MedAtlas SeaDataNet ASCII and CF-compliant netcdf formats are available via SEANOE repository at: https://www.seanoe.org/data/00828/94008/

Specifications Table

**Table:** 

Subject	Oceanography
Specific subject area	Hydrographic data collected in the Ría de Vigo and adjacent shelf, Northwest Iberia, (42° 7.8’ - 42° 13.3’ N, 8° 47.7’ - 9° 7.5’ W)
Type of data	Hydrographic Figure
How the data were acquired	CTD data were collected on a biweekly to monthly schedule from August 1987 to September 2020 along a transect from the interior of Ría de Vigo to the mid-outer shelf. These data were acquired using MARK III, Sea-Bird Scientific 19 SeaCAT, SBE 9 Plus or SBE 25 Sealogger CTD (conductivity, temperature, depth) profilers. They were processed using Sea-Bird Scientific's Seasoft software package, and subsequently quality controlled with both ctdcheck software and Ocean Data View software [1,2]
Data format	Raw Filtered
Description of data collection	This dataset consists of both MedAtlas SeaDataNet ASCII files and a CF-compliant NetCDF file containing 1-dbar interpolated and quality-controlled hydrographic profiles for each cruise. It also includes an example of a Python script that show how to open the netcdf file, read and plot variables, and derive potential density anomalies, among others.
Data source location	· Institution: Centro Oceanográfico de Vigo, Centro Nacional Instituto Español de Oceanografía (IEO, CSIC) · City/Town/Region: Vigo · Country: Spain · Latitude and longitude for collected samples/data: St.15., 42° 13.3’N, 8° 47.7’W, depth 29 m St.1, 42° 12.8’N, 8° 51.0’W, depth 39 m St.3., 42° 8.5’N, 8° 57.5W, depth 97 m St.5., 42° 7.8’N, 9° 7.5’W, depth 148 m
Data accessibility	Repository name: SEANOE. Sea Scientific Open Data Publication. Data identification number: https://doi.org/10.17882/94008 Direct URL to data: https://www.seanoe.org/data/00828/94008/
Related research article	Otero, P., A. Cabrero, F. Alonso-Pérez, J. Gago and E. Nogueira, 2023. Temperature and salinity trends in the northern limit of the Canary Current Upwelling System. Science of the Total Environment. Volume 901, https://doi.org/10.1016/j.scitotenv.2023.165791

## 1 Value of the Data

• Until September 2020, the Radial monitoring program has been the longest (34 years) high frequency sampling (below seasonal scale) based on CTD profiles in the area of the Ría de Vigo and its adjacent shelf, becoming the main tool to examine local hydrographic changes, as well as to understand the connection between changes in ocean climate and the functioning and structure of the ecosystem [3,4].
• The observations presented here, averaged at 1-dbar depth intervals, with quality control processing, are an invaluable source of information for researchers wishing to study topics with potential impact on the region's blue economy, such as long-term trends in hydrographic variables, detection of marine heat waves, ocean acidification, or early detection of harmful algal blooms (HABs) [5,6].
• The time series has suffered interruptions between 1987 and 1994, due to vessel availability at that time. From this date onwards, it has 27 years of biweekly and monthly observations that allow studying the variability of the system in response to wind changes, the connection with atmospheric teleconnection indices, as well as the system response on monthly, seasonal, interannual and decadal scales.
• Data can be downloaded in various standardized formats (SeaDataNet MedAtlas and NetCDF formats) facilitating reuse by the scientific community.

## 2 Objective

Part of the observational data from the Radiales program are freely available in the SeaDataNet marine data infrastructure. However, these data are scattered throughout the infrastructure, and due to the various technicians and researchers responsible for the data over the years of the monitoring program, a homogeneous processing of the data cannot be guaranteed. For this reason, the complete data have been reprocessed following unique criteria, subjected to common quality control tests, supervised one by one, and grouped into a single data set. In addition, an attempt has been made to simplify the dataset in a way that facilitates reuse by the marine scientific community: the data have been averaged at equal depth intervals, the best cast was kept in the dataset, when there are two duplicate sensors the data set retains the most reliable one, standard variable names have been used and a quality control flag was added to each individual record.

## 3 Data Description

**Fig. 1:**
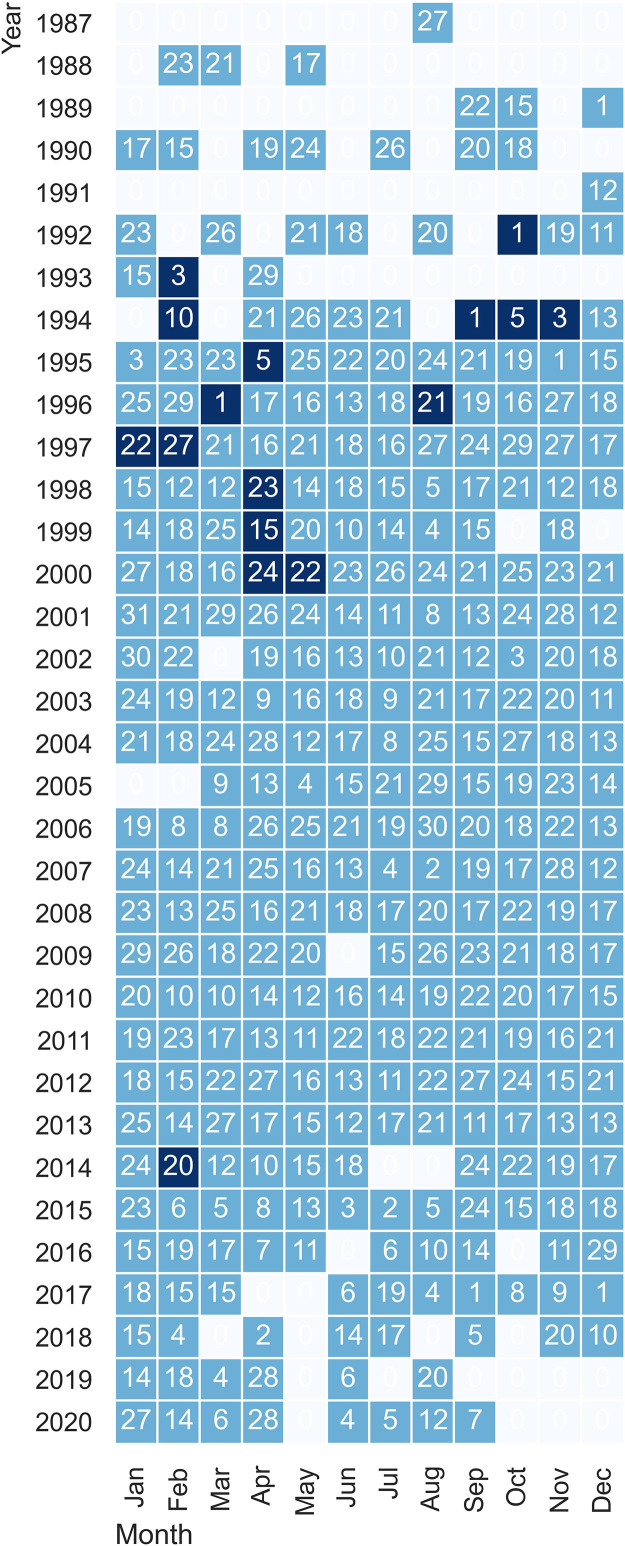
Schedule with the total number of campaigns that make up the dataset, indicating whether there were none (blank), one (light blue) or two campaigns (dark blue) per month. The number indicates the day on which the first campaign of the month took place.

Pressure, temperature and salinity data with their associated quality flags from the Vigo section of the monitoring program “Oceanography Time Series in Northern Spain” (RADIALES; https://www.seriestemporales-ieo.net/) are available in the following repository: https://www.seanoe.org/data/00828/94008/. The compressed archive contains text files under the SeaDataNet MedAtlas format, a self-describing ASCII file specifically designed for describing vertical profiles (http://en.data.ifremer.fr/All-about-data/Data-management/Formats/MedAtlas-Format). Every file is named under the following convention *CTD_29PPYYYYMMDD_000SS.dat*, being *29* the IOC country code, *PP* the ICES code of the platform (*JN* for RV Jose María Naváz, *RM* for B/O Ramón Margalef and *AV* for B/O Ángeles Alvariño), *YYYYY* the year, *MM* the month, *DD* the day and *SS* one of the 4 possible stations (01, 03, 05 or 15), not being necessary that all four stations coexist for the same sampling day (Fig. 1). Additionally, the dataset contains a NetCDF file that follows the CF 1.7. (Climate and Forecast) metadata conventions to simplify the access to the data through Thredds server and OpenDAP protocols as well as fast loading in Ocean Data View software.

## 4 Experimental Design, Materials and Methods

**Fig. 2:**
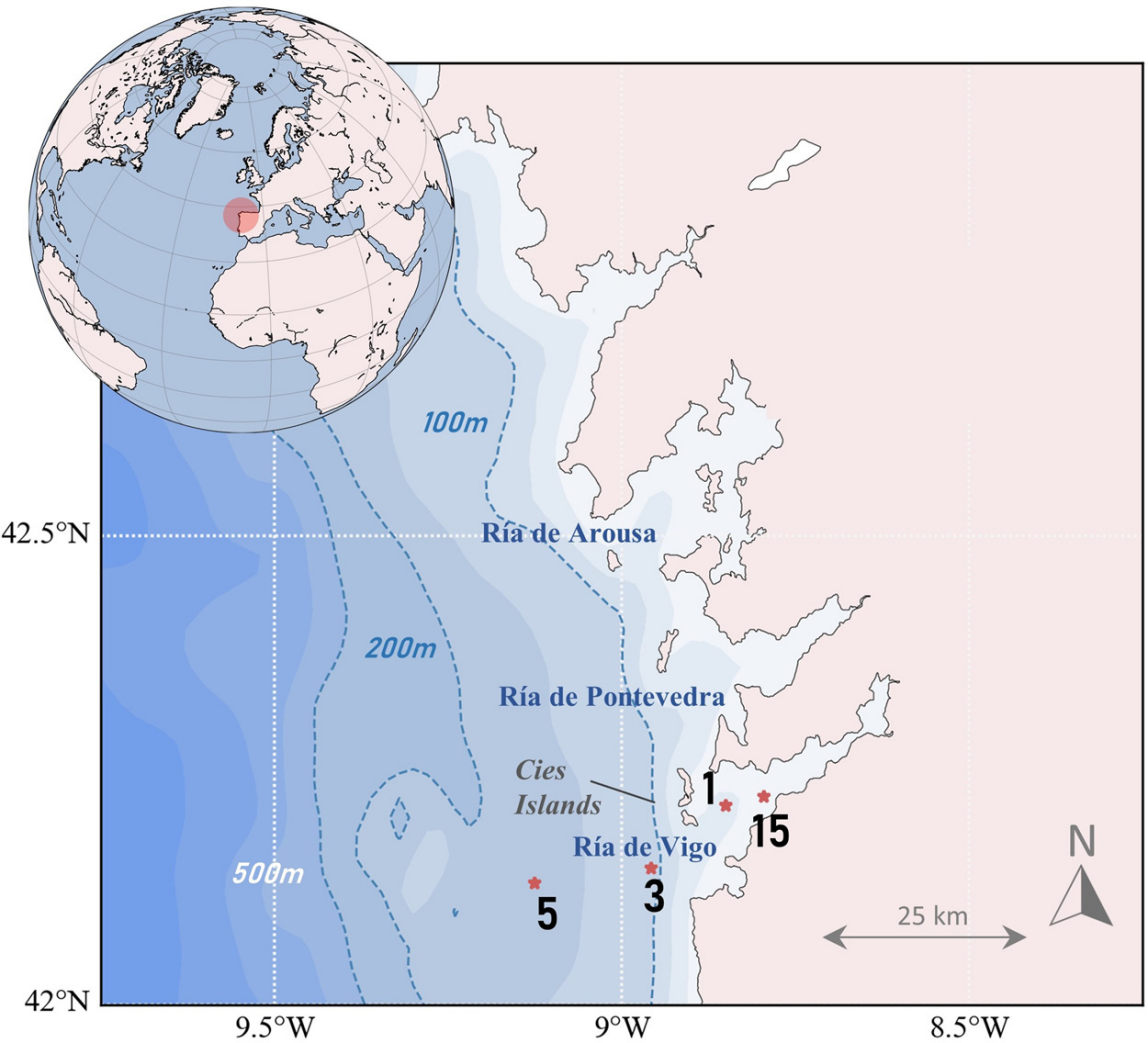
Locations of the CTD stations. The 100 m, 200 m and 500 m isobaths are shown.

The dataset consists of Conductivity–Temperature–Depth (CTD) vertical profile records, made along a section formed by four stations running from the middle of the Ría de Vigo to the mid-outer continental shelf following the route of maximum slope (Fig. 2): a station in the ria closer to the Vigo city (St.15. ., 42° 13.3′N, 8° 47.7′W, depth 29 m) a station in the middle of the ria (St.1, 42° 12.8′N, 8° 51.0′W, depth 39 m), an inner-shelf station located off the southern mouth of the ria (St.3, 42° 8.5′N 8° 57.5W, depth 97 m) and finally a station in the mid-outer shelf (St.5, 42° 7.8′N 9° 7.5′W, depth 148 m). This transect, known as Coastal Ocean Observing System ‘Radial de Vigo’, is part of a set of cross-shore samplings of the Spanish shelf in the frame of the RADIALES monitoring program, carried out by the Centro Nacional Instituto Español de Oceanografía. Data were collected for 34 years, from August 1987 to September 2020, when the series was interrupted due to vessel availability issues. This means a total of 337 campaigns with a monthly frequency, although during some periods the sampling was done twice per month, for example between 1994 and 1995 (Fig. 1). The first years only stations 1, 3 and 5 were sampled; the innermost station, station 15, started to be sampled in January 1997. Subsequently, the outermost station was interrupted in August 2006. On certain occasions, rough sea conditions have been responsible for reducing the number of stations planned for sampling or splitting the sampling into two different days. However, it is stations 1 and 3 that have the longest series. Until June 2017, the vessel RV J.M. Naváz was used as the sampling platform. From that date onwards, sampling was carried out on board the B/O Ramón Margalef and B/O Ángeles Alvariño, depending on their availability.

Data were obtained with one of the following profilers: MARK III, Sea-Bird Scientific 19 SeaCAT or Sea-Bird Scientific 25 Sealogger CTD (Conductivity–Temperature–Depth) in self-contained, or with Sea-Bird Scientific 19 plus in conjunction with the SBE 11 plus deck unit. The profilers were associated with other auxiliary sensors, to measure parameters such as dissolved oxygen, pH, turbidity, fluorescence, light (PAR) or light transmission, however, the suite of these parameters does not possess the robustness and continuity of the thermohaline parameters presented here.

**Fig. 3:**
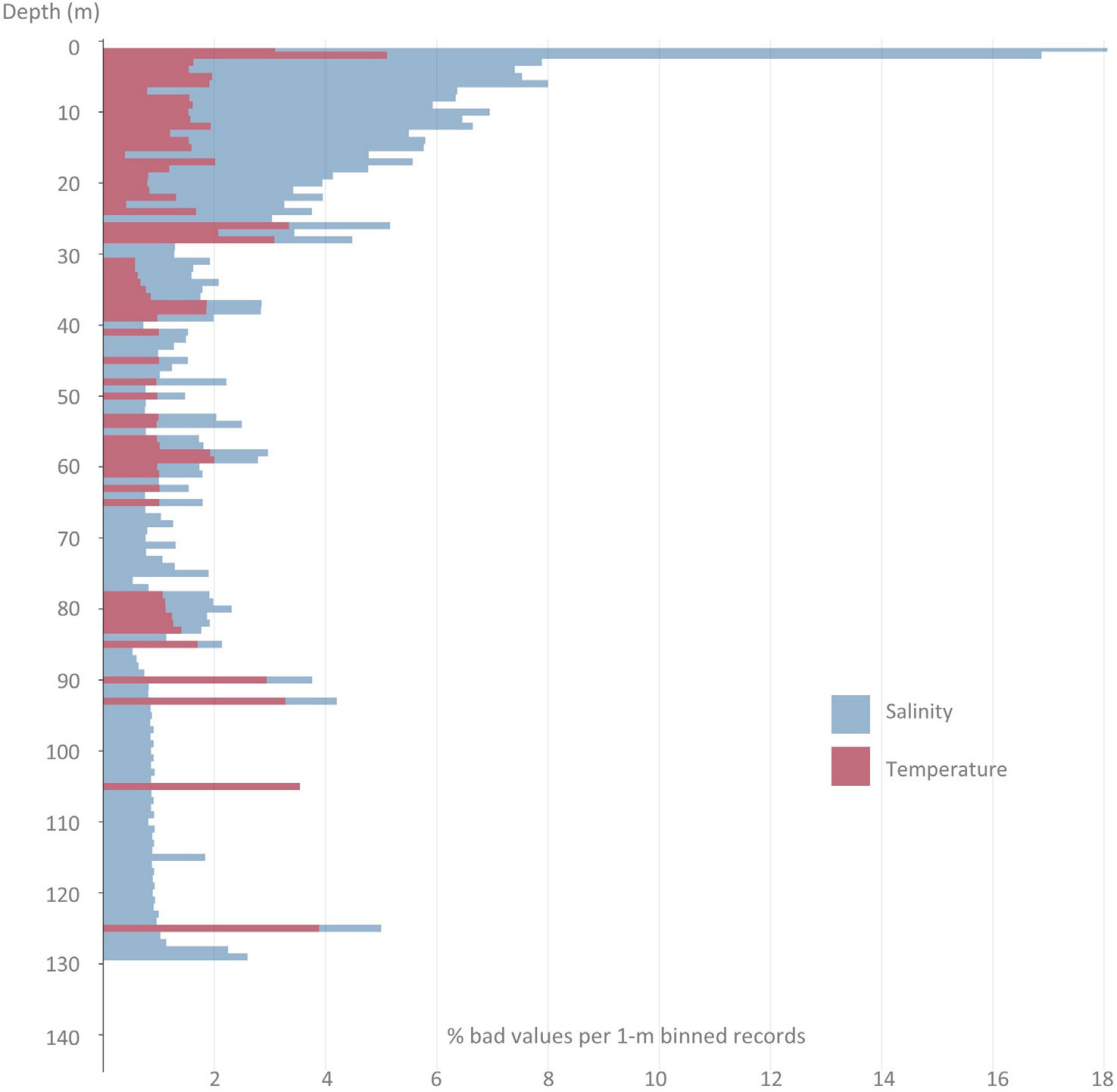
Relative amount of ‘bad’ or ‘probably bad’ flags (%) on 1-m binned temperature and salinity records for the complete dataset.

**Fig. 4:**
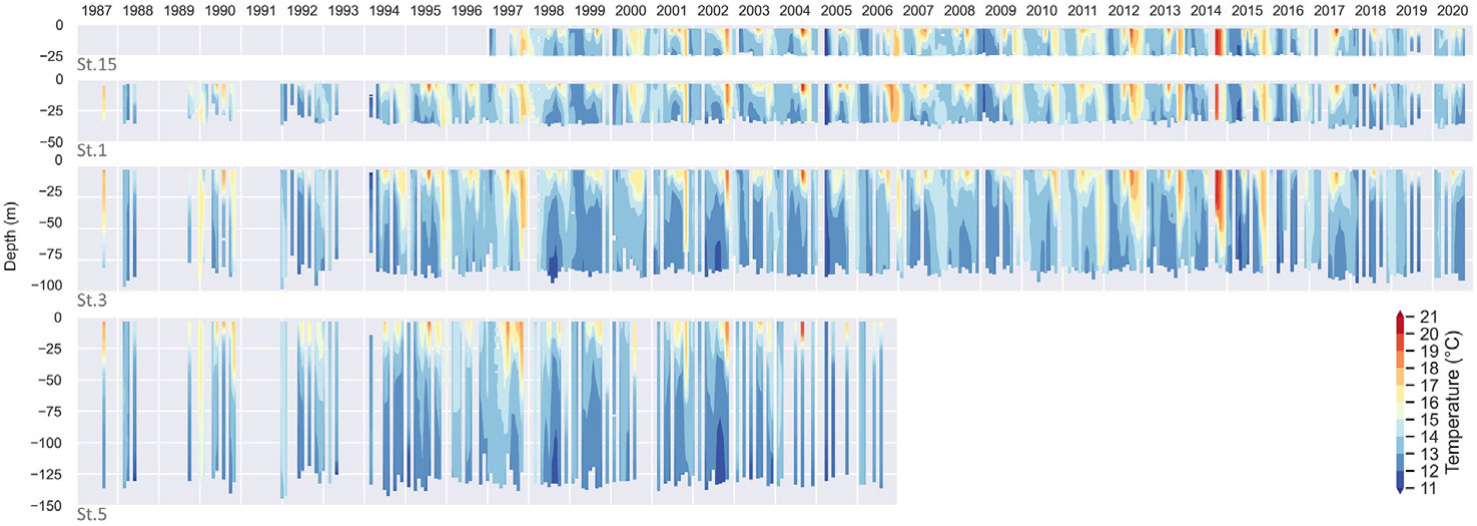
Temperature timeseries for the complete dataset from innermost (top) to outermost (bottom) station.

**Fig. 5:**
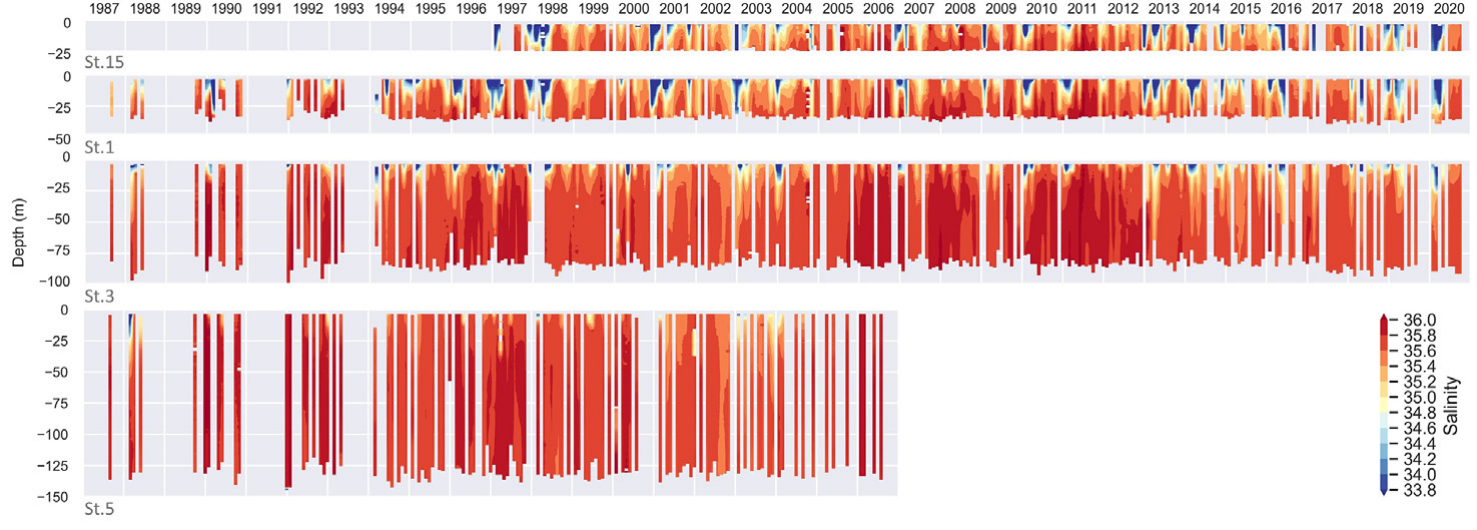
Salinity timeseries for the complete dataset from innermost (top) to outermost (bottom) station.

The various profilers used, as well as the different processing techniques applied, have led to the creation of this dataset in which the whole set of 971 profiles (St.1: 314; St.3: 292; St.5: 116; St.15: 249) has been reviewed and reprocessed under the same criteria. The data for each campaign were processed with Sea-Bird Scientific's Seasoft software package. Temperature and conductivity profiles were aligned and corrected to eliminate any possible drift, spikes were detected, cell thermal mass corrected, pressure reversals removed, practical salinity derived and data averaged in 1-dbar bins. Automatic quality control was applied with the use of the CTDCheck software [1]. The software parses CTD files and splits the profile to prioritize downcast, check the depth, geographical position and time, rename parameters according to common vocabularies, check the pressure and apply different quality tests (e.g., compare with climatology, regional values and density inversion among others). Most of the tests performed by the application reproduce the procedure recommended by the GTSPP [7] and SeaDataNet manuals [8]. Depending on the success of the test, a flag is assigned to each individual record (‘good’, ‘probably good’, ‘probably bad’, and ‘bad’), and also an overall flag to each parameter and to the entire profile. Temperature (salinity) data classified as ‘bad’ or ‘probably bad’ accounted for 1.1 % (3.8 %) of the total records, being the 22.4 % (24.5 %) of them located in the first 5 m of the water column. If we focus on the first 5 m of the water column, St.1 has the higher amount of bad temperature (3.8 %) and salinity (14 %) records, followed by St.15, St.3 and St.5 (Table 1); the inner and shallower the profile, the higher the proportion of ‘bad’ or ‘probably bad’ flags in the entire profile. Fig. 3 shows the relative distribution per depth (1-m bins) of ‘probably bad’ and ‘bad’ temperature and salinity records. It is important to note that sampling from mid-size regional vessels (as the case of *B/O Ramón Margalef* and *B/O Ángeles Alvariño*) or with rough sea conditions, make it difficult the initial tempering of the probes in near-surface waters. For this reason, it is sometimes necessary to dispense with the data closest to the surface, especially in the first 5 m. Figs. 4 and 5 shows the complete time series of valid data for temperature and salinity, respectively. These figures can be reproduced in Python with the script distributed from the code repository: https://github.com/PabloOtero/radial_paper. This code can be easily modified to zoom in on specific dates, as well as compute the potential density anomaly using the standard TEOS-10 formula [9].

**Table 1:** Relative amount of ‘bad’ or ‘probably bad’ flags (%) on temperature and salinity records for each station computed considering the entire profile or just the first 5 m of the water column.

	Temperature				Salinity			
	St.15	St.1	St.3	St.5	St.15	St.1	St.3	St.5
Entire profile	2.2	1.7	0.8	0.6	8.7	7.2	2.1	1.9
First 5 m	2.8	3.8	1.8	1.3	10.9	14.0	9.3	8.5

## Ethics Statement

None.

## CRediT authorship contribution statement

**Pablo Otero:** Conceptualization, Methodology, Software, Visualization, Writing – original draft. **Águeda Cabrero:** Methodology, Data curation, Validation, Writing – review & editing. **Fernando Alonso-Pérez:** Methodology, Data curation, Writing – review & editing. **Gerardo Casas:** Methodology, Data curation, Writing – review & editing. **Jesús Gago:** Resources, Writing – review & editing. **Enrique Nogueira:** Resources, Writing – review & editing, Project administration.

## Data Availability

Temperature, conductivity and salinity data of the RADIALES Program, Vigo, 1987–2020 (Original data) (Seanoe).
